# Fracture Behavior of Bio-Inspired Functionally Graded Soft–Hard Composites Made by Multi-Material 3D Printing: The Case of Colinear Cracks

**DOI:** 10.3390/ma12172735

**Published:** 2019-08-26

**Authors:** Mohammad J. Mirzaali, Alba Herranz de la Nava, Deepthi Gunashekar, Mahdyieh Nouri-Goushki, Eugeni. L. Doubrovski, Amir A. Zadpoor

**Affiliations:** 1 Department of Biomechanical Engineering, Faculty of Mechanical, Maritime, and Materials Engineering, Delft University of Technology (TU Delft), Mekelweg 2, 2628 CD Delft, The Netherlands; 2 Faculty of Industrial Design Engineering (IDE), Delft University of Technology (TU Delft), Landbergstraat, 15, 2628 CE Delft, The Netherlands

**Keywords:** multi-material 3D printing, fracture toughness, hard–soft interfaces, functionally graded materials

## Abstract

The functional gradient is a concept often occurring in nature. This concept can be implemented in the design and fabrication of advanced materials with specific functionalities and properties. Functionally graded materials (FGMs) can effectively eliminate the interface problems in extremely hard–soft connections, and, thus, have numerous and diverse applications in high-tech industries, such as those in biomedical and aerospace fields. Here, using voxel-based multi-material additive manufacturing (AM, = 3D printing) techniques, which works on the basis of material jetting, we studied the fracture behavior of functionally graded soft–hard composites with a pre-existing crack colinear with the gradient direction. We designed, additively manufactured, and mechanically tested the two main types of functionally graded composites, namely, composites with step-wise and continuous gradients. In addition, we changed the length of the transition zone between the hard and soft materials such that it covered 5%, 25%, 50%, or 100% of the width (*W*) of the specimens. The results showed that except for the fracture strain, the fracture properties of the graded specimens decreased as the length of the transition zone increased. Additionally, it was found that specimens with abrupt hard–soft transitions have significantly better fracture properties than those with continuous gradients. Among the composites with gradients, those with step-wise gradients showed a slightly better fracture resistance compared to those with continuous gradients. In contrast, FGMs with continuous gradients showed higher values of elastic stiffness and fracture energy, which makes each gradient function suitable for different loading scenarios. Moreover, regardless of the gradient function used in the design of the specimens, decreasing the length of the transition zone from 100%*W* to 5%*W* increased the fracture resistance of FGMs. We discuss the important underlying fracture mechanisms using data collected from digital image correlation (DIC), digital image microscopy, and scanning electron microscopy (SEM), which were used to analyze the fracture surface.

## 1. Introduction

Functionally graded materials (FGMs) are (multi-phase) composites with spatially varying material properties designed to satisfy specific requirements (e.g., mechanical, thermal, or electrical [1,2,3]). These inhomogeneous materials have several applications in various disciplines, such as aerospace and biomedical engineering [4]. Examples are thermal barrier coatings [5], piezoelectric [6] and thermoelectric devices [7], non-uniform pressurized cylinders [8], (dental) implants [9], biocompatible graded hydrogels [10], soft robotics [11], and tissue engineering scaffolds [12]. In addition to these engineering applications, functional gradients frequently occur in nature, including hard tissues such as bone and tooth [13]. Such functional gradients in the chemical composition of natural materials mainly appear through the selective occurrence of two elementary constituents (i.e., hard (inorganic) and soft (organic) building blocks [14,15]). This usually involves different length scales (i.e., from nano to micro) [16] and a combination of various nano- or micro-architectures (e.g., bricks-and-mortars) [17,18].The incorporation of these structural features in biological materials results in specific toughening mechanisms. The concept of functional gradients is, therefore, one of the design motifs that has its roots in nature [19] and can be implemented in the design of advanced functional materials with properties not achievable using homogenous materials.

One of the advantages of FGMs over traditional composites, such as layered composites, is that abrupt hard–soft interfaces can effectively be eliminated in FGMs. Large discrete zones between two distinct (hard and soft) mediums in traditional layered composite structures create stress concentrations that can consequently decrease the structural integrity in the long term. The high-stress localizations at hard–soft interfaces make these layered composites very susceptible to crack initiation and delamination [20]. In contrast, advanced FGMs circumvent the interface problems and, thus, demonstrate a better performance under critical service conditions, including a longer lifetime [2,3,4,20]. Advanced FGMs can also provide the freedom to create materials with tailored properties designed for specific functions. That makes advanced FGMs an appropriate candidate to be used in many high-tech industries (e.g., biomedical, aerospace, and automotive). As a result, it is important to understand the mechanical properties and fracture behavior of advanced FGMs before putting them to use.

The recent progress in additive manufacturing (AM, = 3D printing) in general and multi-material AM, in particular, has provided new, unparalleled opportunities for the fabrication of arbitrarily complex and precisely controlled FGMs. Previous attempts at the fabrication of FGMs have been primarily focused on ceramic/metal systems [1,21,22] and functionally graded metallic composites fabricated by AM techniques such as selective laser melting (SLM) [23,24] and laser deposition processes [25,26]. The fabrication process of polymer FGMs has been limited and challenging, given the limitations of the conventional manufacturing methods. During the last few years, advanced multi-material additive manufacturing techniques that allow for controlling the deposited material at the voxel level (i.e., length scales in the range of a few tens of micrometers) have been emerging through material jetting processes [27]. Using these emergent techniques, one can precisely control the spatial distribution of mechanical properties in three dimensions at very small scales [28,29,30,31]. These manufacturing methods can be used for the creation of not only heterogeneous, but also graded, structures in volume. Such implementations can also generate new opportunities in the fabrication of advanced FGMs as structural materials with new forms of (multi-) functionalities and properties.

FGMs will face complex three-dimensional states of stress when used in practical applications. From the fracture mechanics viewpoint, this could result in the propagation of cracks existing in different directions either in the hard or the soft material, or at the interface of the hard and soft materials. In order to understand the fracture behavior of FGMs in the actual three-dimensional states of stress, it is important to study the different possible cases separately. With the help of advanced fracture mechanics models, multi-modal fracture properties in an arbitrary direction can then be determined by combining the properties measured for single fracture modes and single crack orientations (with respect to the gradient direction). Here, we focus on the crack propagation from a hard to soft phase. In addition to the general three-dimensional case mentioned above, there are specific engineering applications for this study case, especially where a hard (coating) layer covers and supports a core or substrate made of a soft material. Examples are ceramic-polymer composites for energy absorption [32,33] or biomedical applications [34,35], superamphiphobic surfaces [36], hydrophobic latexes [37], and flexible electronics [38,39,40,41]. Such bi-material examples are often susceptible to cracking at the hard (coating) layer due to the stress induced by the deformations of the soft substrate [42] or due to other tribological behaviors [43]. As a result, understanding the crack propagation in composites with varying fracture properties is of high importance, as the rate and type of crack propagation in two dissimilar mediums may be quite different. Additionally, it is unclear how introducing different gradient functions (e.g., continuous or discontinuous) at the interface of the hard–soft connection, as well as the size of such transition zone, can influence the fracture properties of FGMs. Therefore, this study aims to elucidate the fracture behavior of FGM with cracks along the gradient direction.

## 2. Material and Methods

**Figure 1:**
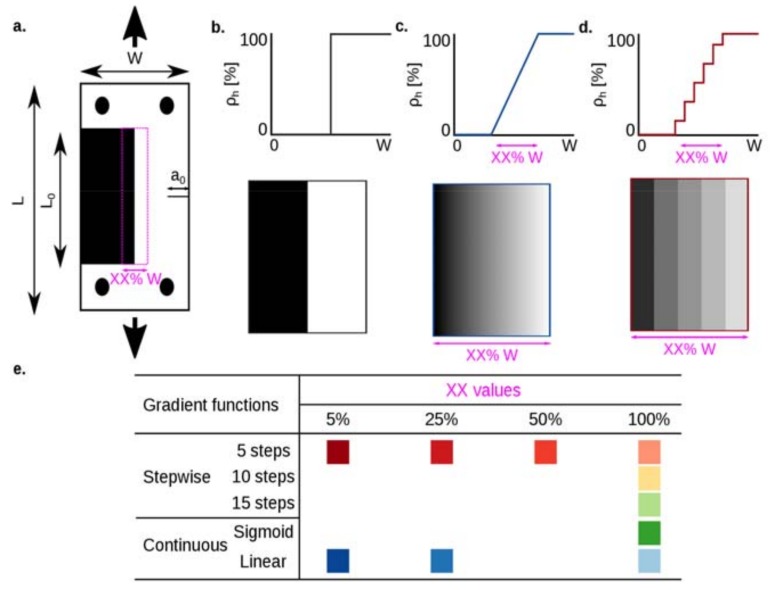
A schematic view of a single-edge notched tensile specimen with an initial crack colinear with the gradient direction (**a**) for non-graded (**b**) and graded (**c**,**d**) specimens. The transition zone between hard and soft phases had two gradient functions, namely, (***i***) step-wise (5 steps, 10 steps, and 15 steps) and (***ii***) continuous (sigmoid and linear). A schematic drawing showing the different gradient functions is presented in Figure S1 of the supplementary document. The size of the transition length between the hard and soft phases was also varied. Three levels were considered for the transition lengths, namely 5%, 25%, and 50% of the width (*W*) of the specimen. The design matrix is presented in (**e**). The white and black pixels respectively represent the hard and soft phases. The geometrical parameters were L=75 mm, W=75 mm, $L_{0}=100$ mm, and $a_{0}=15$ mm.

Here, we studied the fracture behavior of functionally graded soft–hard composites with a pre-existing crack colinear with the gradient direction. Towards this aim, we designed, additively manufactured, and mechanically tested ten types of single-edge notched tensile specimens (Figure 1, and Figure S1 of the supplementary document). The specimens were divided into the following main groups: (1) those with abrupt hard–soft connections without a gradient (Figure 1a and Figure S1a of the supplementary document); (2) three variations of step-wise graded structures, 5-steps (Figure S1b of the supplementary document), 10-steps (Figure S1c of the supplementary document), and 15-steps (Figure S1d of the supplementary document); and (3) continuously graded structures with two different gradient functions, namely sigmoid (Figure S1e of the supplementary document) and linear (Figure S1f of the supplementary document) functions. In groups (2) and (3), the graded functions covered the entire width of the specimens (i.e., 100%*W*). Examples of the 3D printed FGMs are presented in Figure S2 of the supplementary document. We also varied the length of the transition zones between the hard and soft materials in the group (4) by considering three lengths (i.e., 5%*W*, 25%*W*, and 50%*W)*, where *W* is the width of the specimens (Figure 1a). Two gradient functions (i.e., 5 steps and linear) were used when the length of the transition zone was changed between 5%*W* and 25%*W* (Figure 1c–e). For the specimens with a transition zone equal to 50%*W*, only a 5-step gradient was considered.

We could not design our single-edge-notched tension specimens based on a relevant American Society for Testing and Materials (ASTM) standard as no standards have been established for such functionally graded materials. We followed the conditions mentioned in two relevant studies, namely [44,45], for the fracture toughness analysis of soft materials. When designing single-edge notched specimens, it is important to consider the geometrical features of the specimen, including the length to width ratio (usually >1), the initial position of the crack, and the minimum out-of-plane thickness of the specimens, to maintain the plane-strain conditions.

These specimens were fabricated using a multi-material additive manufacturing technique (Objet350 Connex3^TM^ 3D printer, Stratasys^®^ Ltd., Eden Prairie, MN, USA) that works based on material jetting. Two commercially available materials, namely VeroMagenta^TM^ (RGD851, shore hardness (D) 83–86) and Agilus30^TM^ Black (FLX985, shore hardness (A) 30–35), were used for the deposition of hard and soft phases, respectively.

For the fabrication of the graded samples, grayscale images with corresponding gradient functions were created. These images were converted into binary bitmap images using a halftoning algorithm and served as the input files for the 3D printer. The gradient functions, therefore, were created by changing the hard volume fraction (the number of white voxels in the binary image) within the transition zone. For example, in the case of a 5-step gradient, we intended to achieve a certain hard volume fraction corresponding to each step. We, therefore, introduced four intermediate hard volume fractions (i.e., 80%, 60%, 40%, and 20%) to include a gradient from a purely hard (100% hard volume fraction) to a purely soft (0% hard volume fraction) material (Figure 1 and Figures S2 and S3 in the supplementary document).

The overall volume fraction of the hard phase in the specimens of all six groups was equal to 50% (see Table S1 in supplementary documents). The initial crack spanned 20% of the width of the sample and was positioned at the hard part of the specimens.

Each specimen had two extra connecting parts made of the same hard phase that were printed together with the samples. Two aluminum grippers and four aluminum pins were also fabricated for attaching the specimens to the mechanical testing machine. Three samples were tested in each group. The geometrical parameters of the specimens are presented in Figure 1a. The out-of-plane thickness of the specimens, t, was 3 mm.

Fracture tests were performed under displacement control (stroke rate = 2 mm/min) using a mechanical testing machine (LLOYD instrument (LR5K) with a 5 kN load cell). At a sampling rate of 20 Hz, the time, force, and displacement were recorded. The normal stress, σ, was defined as the ratio of the force, F, to the effective cross-sectional area, $A_{0}=t\times \left(W-a_{0}\right)$, of the specimens. The strain, ε, was defined as the ratio of the displacement in the longitudinal direction, $u_{y}$, to the initial free length between the grippers, L. The stiffness, E, was calculated using a moving regression algorithm with a bounding box of 0.2% strain to measure the stiffest part of the loading. The fracture stress, $\sigma_{f}$, was defined as the maximum stress. The fracture toughness, U, was calculated from the numerical integration of the area under the stress–strain curve until the end of the test (final fracture). The final strain, $\varepsilon_{f}$, was defined as the maximum strain at the end of the test (rupture point). The mechanical properties calculated from the experimental results are visually presented in Figure S3a (see the supplementary document).

Full-field strain measurements were performed for the specimens with both stepwise (i.e., 5 steps) and continuous (i.e., linear) gradients and a length of the transition zone equal to 100%*W* using the digital image correlation (DIC) technique [46,47]. A commercial DIC system including two digital cameras (4 MP with CMOS chip) and the associated software (Vic-3D 1, Correlated Solutions, SC, USA) was used to determine the strain distribution. For this purpose, applied on one side of the specimen, a speckle pattern was created by randomly spraying black dots on a white background.

The fracture surfaces of samples were analyzed using digital microscopy (Keyence^®^ vhx-5000) with a zoom lens (VH-Z20T) at 200× magnification. Scanning electron microscopy (SEM-JEOL, Tokyo, Japan) with 10 kV at 35,000× magnification was also used to analyze the fracture surface characteristics of representative specimens in more detail. All specimens were gold-sputtered using a sputter coater (JFC-1300, JEOL, Tokyo, Japan) for 18 s with a coating thickness of 2 nm before imaging.

The statistical analyses were performed in R [48]. An ANOVA (analysis of variance) with a post-hoc Tukey HSD (honestly significant difference) test was performed to compute the significant differences between the fracture properties of different groups.

## 3. Results and Discussion

**Figure 2:**
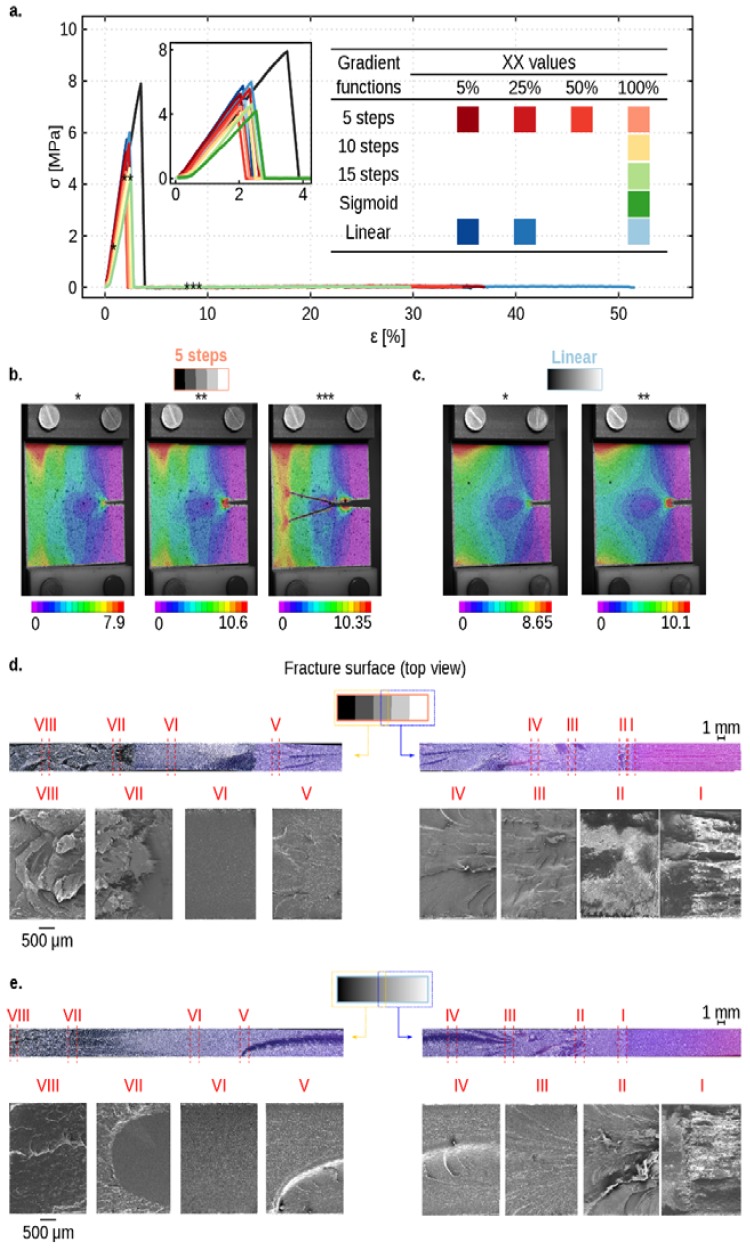
(**a**) Typical stress–strain curves of specimens with graded or non-graded interfaces. The inset shows a magnified view of the stress–strain curves until 4% strain. Full-field strain measurement using digital image correlation (DIC) of specimens with five steps (**b**) and linear (**c**) transition functions. DIC images show von Mises strain before maximum load (*), at the maximum load (**), and after maximum load (***). Digital microscopy images (200× magnifications) and the corresponding scanning electron microscopy (SEM) (35,000× magnifications) analyses of the fracture surface of the specimens with five steps and linear transition function are presented in sub-figures (**d**) and (**e**), respectively. The SEM images show the zoomed-in images of eight regions of structures. The scale bars for the digital microscopy images are on top of the corresponding images and underneath the SEM images. The hard phase is in white pixels, while the soft phase is in black. The size of the transition length was 100%*W* for the specimens in subfigures (**b**–**e**).

The stress–strain behavior of all samples followed the same trend (Figure 2a). Their curves consisted of two stages. In the first stage, the force linearly increased as the specimen was stretched. This phase continued until a brittle fracture occurred. This resulted in a sudden drop in the force (stress). The initial fracture was swift and started at small strains (between 3% and 5% longitudinal strains). Later, in the second stage, the level of force remained almost constant until the end of the test, where the specimen broke apart (Figure S3b of the supplementary document).

The cracks started to propagate from the hard side of the structure. They were sharp before the initial fracture in the hard region. However, crack blunting was observed when cracks started to propagate in the soft part. Crack bridging was observed in the cases with sharp hard–soft interfaces and those with stepwise transitions (Figure 2b), regardless of the length of the transition zone. The positions where crack bridging started were usually close to the center of the specimens and a few millimeters away from the initial crack. In the cases with a continuous gradient, cracks propagated in the structures without significant bridging (Figure 2c). However, decreasing the transition zone from 100%*W* to 5%*W* resulted in similar crack bridging that was already observed in the hard–soft abrupt transitions and 5-step gradients.

From DIC images, a high-strain region in front of the crack tip was observed at the maximum force (Figure 2b,c). Strain localization also existed from the far field to the crack tip at the interface of soft parts of the specimens and hard-connecting regions. The size of this region was smaller for the samples with a linear hard–soft transition (Figure 2c). 

In all cases, micro-cracks were seen near and perpendicular to the fracture surface (Figure 2d,e). These micro-cracks were the result of an initial brittle fracture and crack bridging in the structures. They usually started in the middle of the fracture surface and did not follow a straight path. They also deviated towards the free surfaces of the structures to reach the surfaces with minimum energy (zones I to III in Figure 2d,e). Such deflections in the crack propagation created semi-elliptical macro-cracks in the transition zones (zone IV and V in Figure 2d,e).

The distribution of these macro- and micro-cracks was high near the locations where initial cracks originated. However, as it moved towards the soft region at the other side of the structure, the number of cracks reduced significantly (zone VI in Figure 2d,e). This showed that when the cracks had entered the transition zones, their driving forces had been reduced, resulting in energy dissipation (zone VII in Figure 2d,e). 

The energy required to open up the cracks in the hard region was significantly higher than the energy needed to open the cracks in the soft regions (Figure 2a). The fracture surface and crack propagation in the soft region were different from the hard region. A high level of strains (around 20% for almost all cases except those with the linear transition) was required to separate the specimens. That created a wavy fracture surface in the soft region (zone VIII in Figure 2d,e) compared to a flat surface in the hard region. The orientations of macro- and micro-cracks in the soft regions were also parallel to the fracture surface (zone VIII in Figure 2d,e).

**Figure 3:**
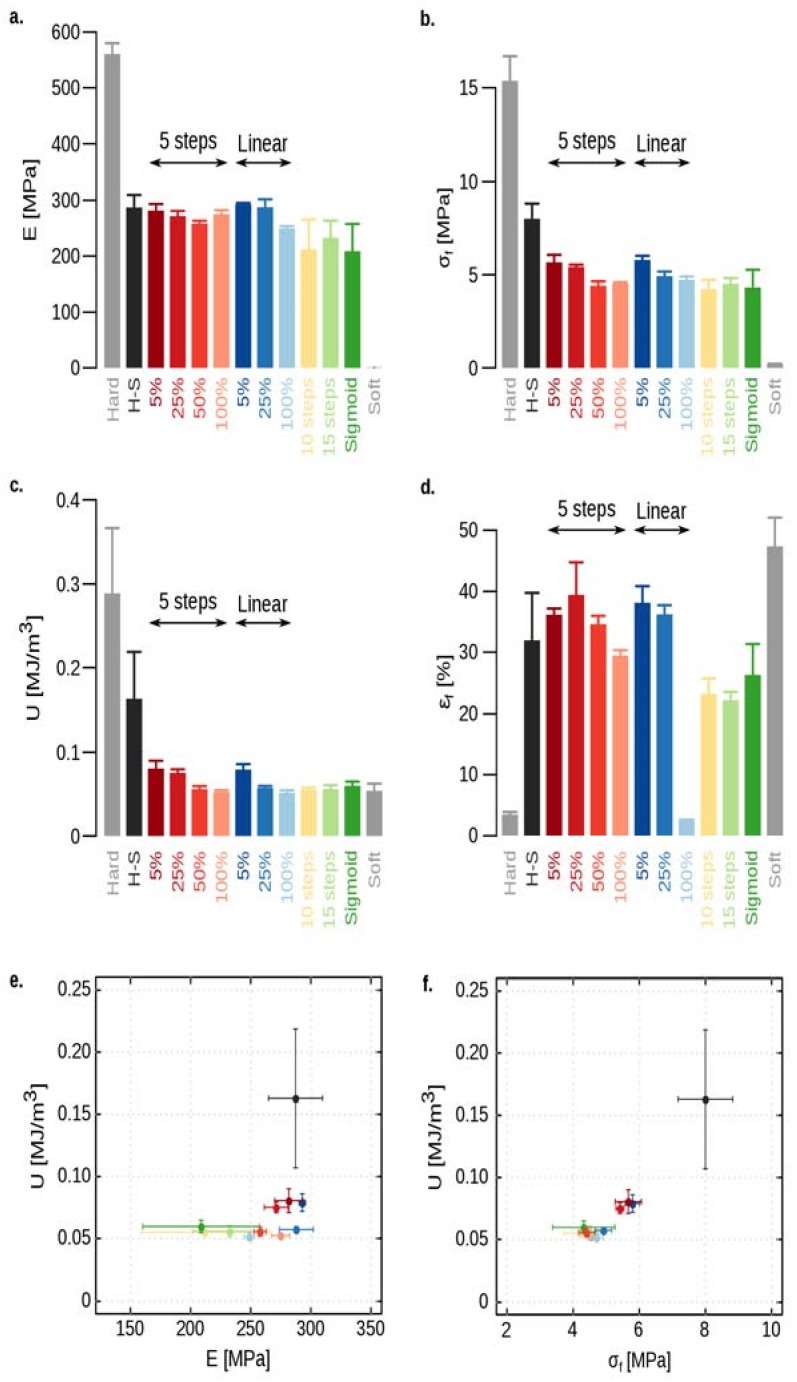
Bar-plots for the comparison of the mechanical properties of the specimens in ten groups, which include elastic stiffness (**a**), fracture stress (**b**), fracture energy (**c**), and final strain (**d**). The statistical parameters of the calculated fracture properties are summarized in Table S2 of the supplementary document. An ANOVA analysis and a post-hoc Tukey honestly significant difference (HSD) test were used to determine whether there was a significant difference between different groups. The comparison tables are presented in Tables S3–S6 of the supplementary document. The Ashby plots compare the fracture energy with elastic stiffness (**e**) and fracture stress (**f**). The plots show the mean and standard deviations for each data point. The fracture properties of the monolithic hard and soft specimens are adopted from [30].

All fracture properties except for the fracture strain of samples with abrupt transitions were higher than those with a gradient transition (Figure 3 and Table S2 of the supplementary document). For the cases with a transition length of 100%*W*, this could be due to the fact that the amount of bulk hard material at the location of the initial crack tip was the greatest for the cases with sharp hard–soft transitions (Table S1 and Figure S1 in supplementary documents). However, the average of hard materials in front of the pre-existing crack was similar within all cases with a transition length of 100%*W* (Table S1 in supplementary documents). The elastic stiffness, fracture stress, and fracture energy of all graded cases are in the same order of magnitude (Figure 3a–c,e,f). It appears that the only parameter that can describe the fracture properties well is the amount of hard material in front of the crack tip and not the types of gradient function. Therefore, for the specimens with lower transition zones (i.e., 5%*W*, 25%*W*, and 50%*W*), we placed the crack tip at the fully hard region and kept the overall hard volume fraction (i.e., $\rho_{h}=50\%$) similar to that of hard–soft abrupt transition (Table S1 in the supplementary document). Comparing these groups showed that decreasing the transition zone increased the fracture properties (i.e., elastic stiffness, fracture stress, and fracture energy). The elastic stiffness of the specimens with no gradient was significantly different from that with 10-steps and sigmoid gradients. However, when the transition zone was smaller (i.e., 5%*W*, 25%*W*, or 50%*W*), no significant difference was observed between the specimens with and without a gradient (Table S3 of the supplementary document). The fracture stress and fracture energy were similar between the groups with different gradient functions, but were significantly different from those of abrupt hard–soft transitions (Tables S4 and S5 of the supplementary document).

We also compared the fracture properties of our FGMs with those of monolithic hard and soft specimens tested previously in [30]. All the fracture properties of abrupt hard–soft specimens and FGMs were within the range of the pure hard and soft materials (Figure 3a–d). All the fracture properties of monolithic hard and soft specimens were significantly different from those of FGMs with or without a gradient (Tables S3–S6 of the supplementary document).

Among the two types of transition functions (i.e., stepwise and continuous), the samples with a continuous gradient showed slightly higher fracture stress (e.g., linear Figure 3b,f), higher fracture energy (Figure 3c,e,f), and higher elastic stiffness (Figure 3a,e), especially for the specimens with the shortest transition zones (i.e., 5%*W*).

The final strain is the only parameter that was significantly lower for the specimens with a linear gradient and transition length of 100%*W* (Figure 3d). As the linear gradient function had a smooth and continuous transition compared to step-wise graded specimens, cracks propagated more rapidly through the linear gradient structure without much resistance (Figure S2 of the supplementary document). In other words, the soft region in step-wise cases behaves as a crack barrier in the structures resisting crack propagations in the soft region. Since the size of the pure soft regions is the smallest for the cases with a linear gradient, the final strain was the lowest (Figure 3d) for those with a transition length of 100%*W*.

The size of the pure soft region for the samples with a sigmoid function was between those having an abrupt hard–soft transition and the step-wise graded structures. This is why the final strain of sigmoid graded structures was higher than that with linear, 15-step, and 10-step gradients, but lower than those with a 5-step gradient and sharp hard–soft transition (Figure 3d). In that sense, the samples with a 5-step gradient showed the best fracture resistance and those with a linear gradient showed the worst. Decreasing the transition length from 100%*W* to 5%*W*, however, highly influenced the fracture strain. Regardless of the type of gradient function at the transition zone, the fracture strain of the specimens with a transition length of 5%*W* was 20% higher than that of the specimens with abrupt hard–soft transitions (Figure 3d, and Table S6 of the supplementary document).

As mentioned before, FGMs in general and polymer FGMs in particular offer great promise, especially when parts are designed to work under severe conditions (e.g., high temperature, abrasive chemical interactions, etc.), which makes their potential applications diverse and numerous. Graded thermal barrier coatings [5] and composite graded patches [49] are some examples of the potential applications.

FGMs are also very promising in tissue engineering, notably for the fabrication of synthesized functionally graded bio-scaffolds [50] for tissue regeneration, as cell responses depend on the mechanical properties of the substrates [51]. Examples are graded porous implants used for repairing bone-cartilage tissue, the regeneration of graded tendon/ligament-to-bone insertions [52], and functionally graded dental implants [9].

FGMs can also be combined with mechanical metamaterials [53,54,55,56,57,58], another class of advanced materials whose mechanical properties originate from their geometrical designs at the micro-scale. This is important, especially when dual-phase (i.e., soft–hard) metamaterials [59,60,61] are considered, as employing the concept of the functional gradient can not only smoothen the stress concentrations present at the interfaces of soft and hard phases, but also be used to add more functionalities to these materials.

## 4. Conclusions

In summary, we studied a specific case of the fracture behavior of FGM, namely for cracks along the gradient direction. Other types of loading, such as impact [62] and crack propagation under fatigue loading, can be conducted to evaluate the response of such FGMs under these loadings. We found that, unlike fracture in other directions, the fracture properties (e.g., fracture strain) that are measured for the cracks that are colinear to the gradient direction appear to improve when the material gradient is non-continuous. FGMs with a continuous gradient, on the other hand, showed slightly higher stiffness and fracture energies. This suggests that the type of gradient function (i.e., continuous or non-continuous) may affect the fracture behavior of FGMs differently, depending on the orientation of the crack with respect to the gradient direction. More importantly, in addition to the type of gradient functions, the length of the transition zone between the hard and soft phases is the most critical parameter influencing the fracture resistance of FGMs to crack growth that needs to be included in the design of these advanced materials.

## Supplementary Materials

The following are available online at https://www.mdpi.com/1996-1944/12/17/2735/s1: Table S1: A comparison of the overall hard volume fractions in different design cases. The average of the hard material fraction in front of the pre-existing crack and the exact amount of the hard material at the crack tip for different designs are also compared, Table S2: Mean ± standard deviation of the fracture properties of FGM specimens with and without gradient as well as the properties of the purely hard and soft materials, Table S3: A comparison of the stiffness, E, between different groups. The table shows *p*-values calculated with ANOVA (analysis of variance) using the *post-hoc* Tukey HSD (honestly significant difference) test. Only significantly different groups are reported. The *p*-values less than 0.01 are shown by (**). (*) means 0.01 < *p*-value < 0.05, Table S4: A comparison of the elastic fracture stress, $\sigma_{f}$, between different groups. The table shows the *p*-values calculated with ANOVA (analysis of variance) using the *post-hoc Tukey HSD* (honestly significant difference) test. Only significantly different groups are reported. The *p*-values less than 0.01 are shown by (**). (*) means 0.01 < *p*-value < 0.05, Table S5: A comparison of the fracture energy, U, between different groups. The table shows the *p*-values calculated with ANOVA (analysis of variance) using the *post-hoc* Tukey HSD (honestly significant difference) test. Only significantly different groups are reported. The *p*-values less than 0.01 are shown by (**). (*) means 0.01 < *p*-value < 0.05, Table S6: A comparison of the fracture strain, $\varepsilon_{f}$, between different groups. The table shows *p*-values calculated with ANOVA (analysis of variance) uing the *post-hoc* Tukey HSD (honestly significant difference) test. Only significantly different groups are reported. The *p*-values less than 0.01 are shown by (**). (*) means 0.01 < *p*-value < 0.05, Figure S1: The distribution of the hard material, $\rho_{h}$, in the functionally graded composites with (a) abrupt hard- soft transition without gradient, step-wise (5-steps (b), 10-steps (c), and 15-steps (d)) and continuous gradients (sigmoid (e) and linear (f)). The geometrical parameters are W=75 mm, and $a_{0}=15$ mm. The exact values for the percentage of the hard material at the the crack tip for different designs are presented in Table S1. The transition length for these specimens were 100%*W*, Figure S2: Photos of the specimens with linear (a), 5-steps (b), 10-steps (c), and 15-steps (d) gradients before and after the fracture tests. The transition length for these specimens was 100%*W*. The pink and black sides are respectively made from purely hard and purely soft phases. Changing the gradient from a non-continuous (i.e., 5-steps) to a continuous (i.e., linear) function resulted in crack deflections, Figure S3: An illustration of the way the mechanical properties were calculated from the typical stress-strain curves of graded/non-graded specimens (a). The propagation of a crack initiated in the hard (pink) phase to the soft (black) phase in a specimen with a 5-steps gradient and a transition length of 100%*W* (b). The initial crack swiftly propagated in the hard region (I-III), demonstrating the hallmarks of a brittle failure. The crack stopped at the soft region (IV) and exhibited a blunting zone at its tip during (V).
