# Supplementary material for: Fracture Behavior of Bio-Inspired Functionally Graded Soft–Hard Composites Made by Multi-Material 3D Printing: The Case of Colinear Cracks
Source: Materials (Basel). 2019 Aug 26;12(17):2735. doi: 10.3390/ma12172735 (PMC6747772; doi:10.3390/ma12172735)
Supplement: Supplementary file 1 [file materials-12-02735-s001.pdf]

materials

Supplementary document to

Fracture Behavior of Bio-Inspired Functionally Graded Soft–Hard Composites Made by Multi-Material 3D Printing: The Case of Colinear Cracks

Mohammad J. Mirzaali [1,*], Alba Herranz de la Nava [1], Deepthi Gunashekar [1], Mahdyieh Nouri-Goushki [1], Eugeni. L. Doubrovski [2] and Amir A. Zadpoor [1]

[1] Department of Biomechanical Engineering, Faculty of Mechanical, Maritime, and Materials Engineering, Delft University of Technology (TU Delft), Mekelweg 2, 2628 CD Delft, The Netherlands

[2] Faculty of Industrial Design Engineering (IDE), Delft University of Technology (TU Delft), Landbergstraat, 15, 2628 CE Delft, The Netherlands

* Correspondence: m.j.mirzaalimazandarani@tudelft.nl; Tel.: +31-15-2783133

Table S1. A comparison of the overall hard volume fractions in different design cases. The average of the hard material fraction in front of the pre-existing crack and the exact amount of the hard material at the crack tip for different designs are also compared.

Specimens	Transition Length [%*W*]	ρ_h [%]	Average of Hard Materials in Front of the Pre-Existing Crack [%]	Percentage of Hard Materials at the Crack Tip [%]
Hard-soft abrupt	0	50.00	37.36	100.00
5 Steps	100	50.16	37.24	74.90
	50	49.65	28.59	100
	25	49.50	27.91	100
	5	49.46	29.94	100
10 Steps	100	49.89	38.65	77.64
15 Steps	100	50.40	38.70	78.43
Sigmoid	100	50.02	38.37	98.43
Linear	100	50.06	39.85	79.61
	25	49.41	28.93	100
	5	49.46	29.94	100

Table S2. Mean ± standard deviation of the fracture properties of FGM specimens with and without gradient as well as the properties of the purely hard and soft materials.

Specimens		E [MPa]	σ_f [MPa]	U [$\frac{MJ}{m^3}$]	ε_f [mm/mm]
Hard		560.72 ± 19.46	15.36 ± 1.34	0.29 ± 0.08	0.03 ± 0.00
Hard-Soft		287.28 ± 22.42	8.00 ± 0.83	0.16 ± 0.06	0.32 ± 0.08
5 steps	5%	281.66 ± 11.79	5.67 ± 0.40	0.08 ± 0.01	0.36 ± 0.01
	25%	271.11 ± 9.82	5.42 ± 0.12	0.08 ± 0.00	0.39 ± 0.05
	50%	257.81 ± 5.05	4.41 ± 0.24	0.06 ± 0.00	0.35 ± 0.01
	100%	274.82 ± 7.49	4.53 ± 0.08	0.05 ± 0.00	0.29 ± 0.01
Linear	5%	292.80 ± 2.71	5.79 ± 0.21	0.08 ± 0.01	0.38 ± 0.03
	25%	287.82 ± 14.11	4.92 ± 0.25	0.06 ± 0.00	0.36 ± 0.02
	100%	249.03 ± 4.24	4.71 ± 0.19	0.05 ± 0.00	0.03 ± 0.00
10 steps	100%	212.07 ± 52.92	4.23 ± 0.49	0.05 ± 0.00	0.23 ± 0.03
15 steps	100%	232.56 ± 30.64	4.51 ± 0.31	0.06 ± 0.00	0.22 ± 0.01
sigmoid	100%	208.76 ± 48.47	4.32 ± 0.94	0.06 ± 0.01	0.26 ± 0.05
Soft		0.69 ± 0.06	0.20 ± 0.01	0.05 ± 0.01	0.47 ± 0.05

Table S3. A comparison of the stiffness, E, between different groups. The table shows p-values calculated with ANOVA (analysis of variance) using the *post-hoc* Tukey HSD (honestly significant difference) test. Only significantly different groups are reported. The p-values less than 0.01 are shown by (**). (*) means 0.01 < p-value < 0.05.

		**Hard**	**Hard-Soft**	**5 Steps**				**Linear**			**10 Steps**	**15 Steps**	**Sigmoid**	**Soft**
				**5%**	**25%**	**50%**	**100%**	**5%**	**25%**	**100%**	**100%**	**100%**	**100%**	
Hard			**	**	**	**	**	**	**	**	**	**	**	**
Hard-Soft											*		*	**
5 steps	5%										*		*	**
	25%													**
	50%													**
	100%													**
Linear	5%										*		*	**
	25%										*		*	**
	100%													**
10 steps	100%													**
15 steps	100%													**
sigmoid	100%													**
Soft														

Table S4. A comparison of the elastic fracture stress, σ_f, between different groups. The table shows the p-values calculated with ANOVA (analysis of variance) using the *post-hoc Tukey HSD* (honestly significant difference) test. Only significantly different groups are reported. The p-values less than 0.01 are shown by (**). (*) means 0.01 < p-value < 0.05.

		**Hard**	**Hard-Soft**	**5 Steps**				**Linear**			**10 Steps**	**15 Steps**	**Sigmoid**	**Soft**
				**5%**	**25%**	**50%**	**100%**	**5%**	**25%**	**100%**	**100%**	**100%**	**100%**	
Hard			**	**	**	**	**	**	**	**	**	**	**	**
Hard-Soft				**	**	**	**	**	**	**	**	**	**	**
5 steps	5%													**
	25%													**
	50%													**
	100%													**
Linear	5%													**
	25%													**
	100%													**
10 steps	100%													**
15 steps	100%													**
sigmoid	100%													**
Soft														

Table S5. A comparison of the fracture energy, U, between different groups. The table shows the *p*-values calculated with ANOVA (analysis of variance) using the *post-hoc* Tukey HSD (honestly significant difference) test. Only significantly different groups are reported. The *p*-values less than 0.01 are shown by (**). (*) means 0.01 < *p*-value < 0.05.

		Hard	Hard-Soft	5 Steps				Linear			10 Steps	15 Steps	Sigmoid	Soft
				5%	25%	50%	100%	5%	25%	100%	100%	100%	100%	
Hard			**	**	**	**	**	**	**	**	**	**	**	**
Hard-Soft				*	*	**	**	*	**	**	**	**	**	**
5 steps	5%													
	25%													
	50%													
	100%													
Linear	5%													
	25%													
	100%													
10 steps	100%													
15 steps	100%													
sigmoid	100%													
Soft														

Table S6. A comparison of the fracture strain, ε_f, between different groups. The table shows *p*-values calculated with ANOVA (analysis of variance) using the *post-hoc* Tukey HSD (honestly significant difference) test. Only significantly different groups are reported. The *p*-values less than 0.01 are shown by (**). (*) means 0.01 < *p*-value < 0.05.

		Hard	Hard-Soft	5 steps				Linear			10 steps	15 steps	sigmoid	Soft
				5%	25%	50%	100%	5%	25%	100%	100%	100%	100%	
Hard			**	**	**	**	**	**	**		**	**	**	**
Hard-Soft										**				**
5 steps	5%									**	**	**		*
	25%									**	**	**	*	
	50%									**	*	*		**
	100%									**				**
Linear	5%									**	**	**		
	25%									**	**	**		*
	100%										**	**	**	**
10 steps	100%													**
15 steps	100%													**
sigmoid	100%													**
Soft														

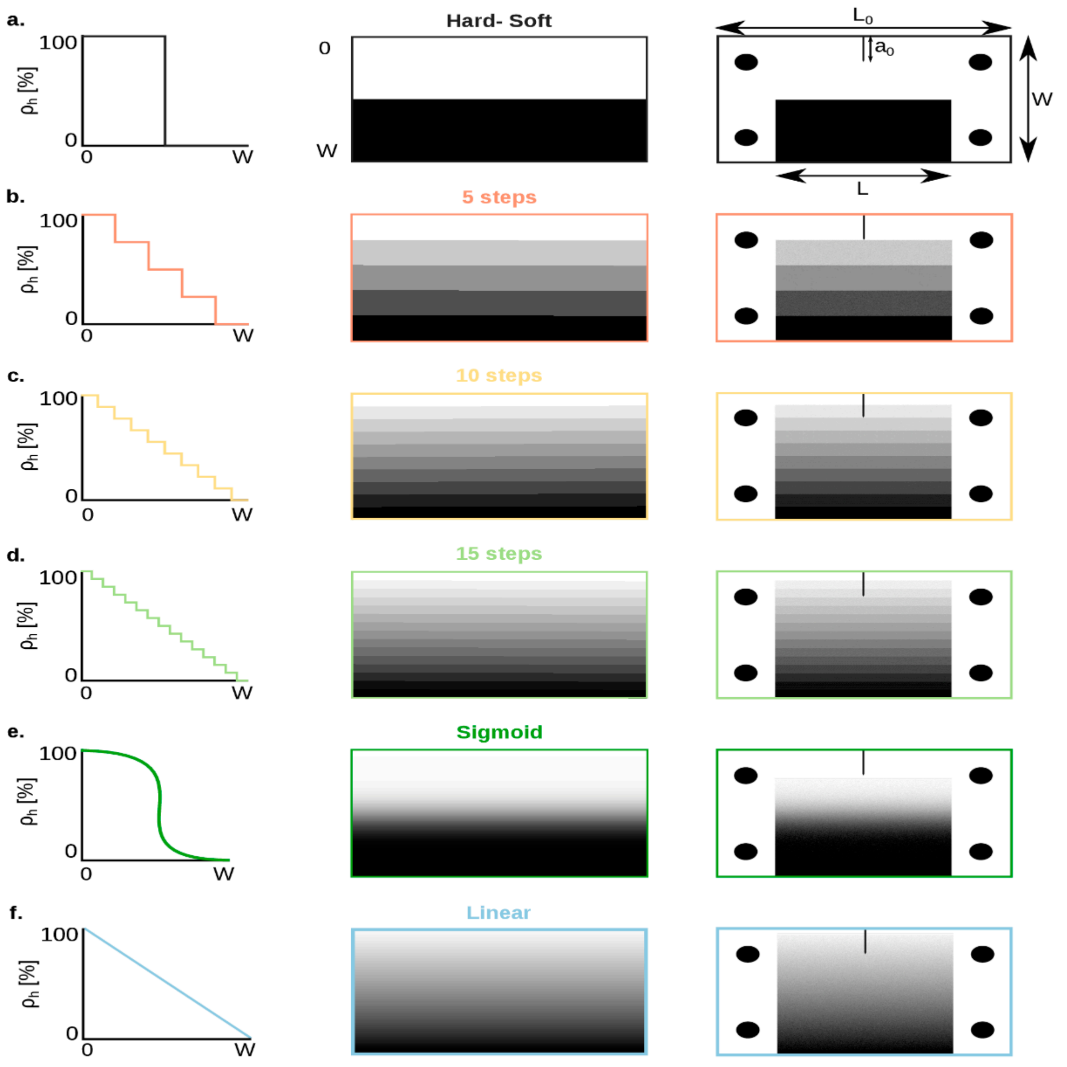

Figure S1. The distribution of the hard material, ρ_h, in the functionally graded composites with (a) abrupt hard- soft transition without gradient, step-wise (5-steps (b), 10-steps (c), and 15-steps (d)) and continuous gradients (sigmoid (e) and linear (f)). The geometrical parameters are $W = 75$ mm, and $a_0 = 15$ mm. The exact values for the percentage of the hard material at the the crack tip for different designs are presented in Table S1. The transition length for these specimens were 100%W.

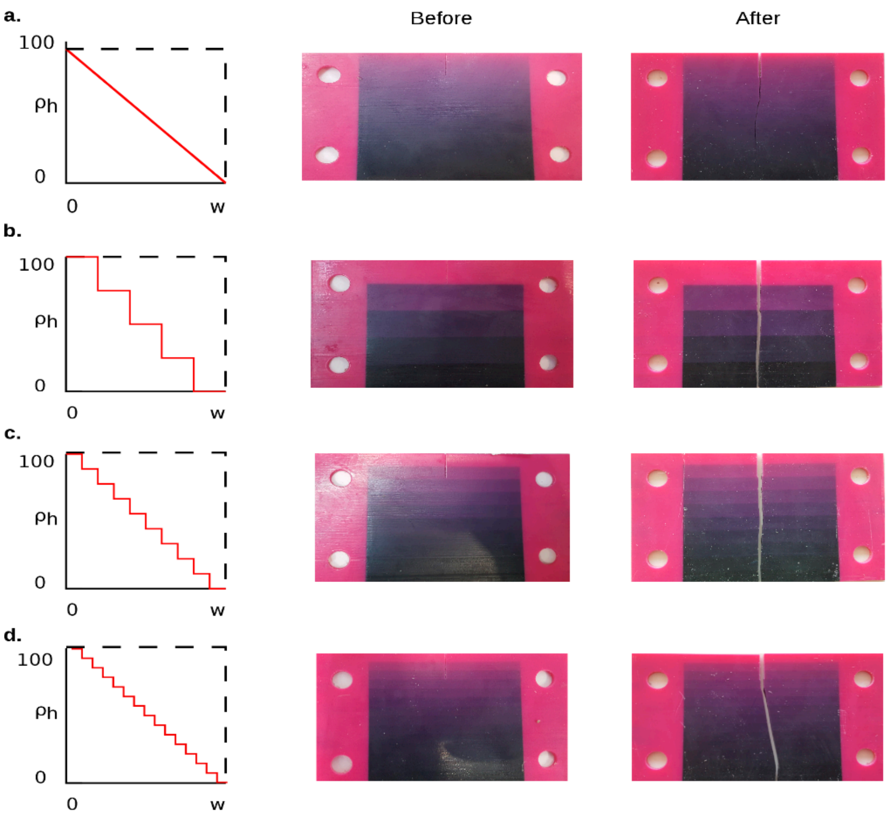

Figure S2. Photos of the specimens with linear (a), 5-steps (b), 10-steps (c), and 15-steps (d) gradients before and after the fracture tests. The transition length for these specimens was 100%*W*. The pink and black sides are respectively made from purely hard and purely soft phases. Changing the gradient from a non-continuous (i.e., 5-steps) to a continuous (i.e., linear) function resulted in crack deflections.

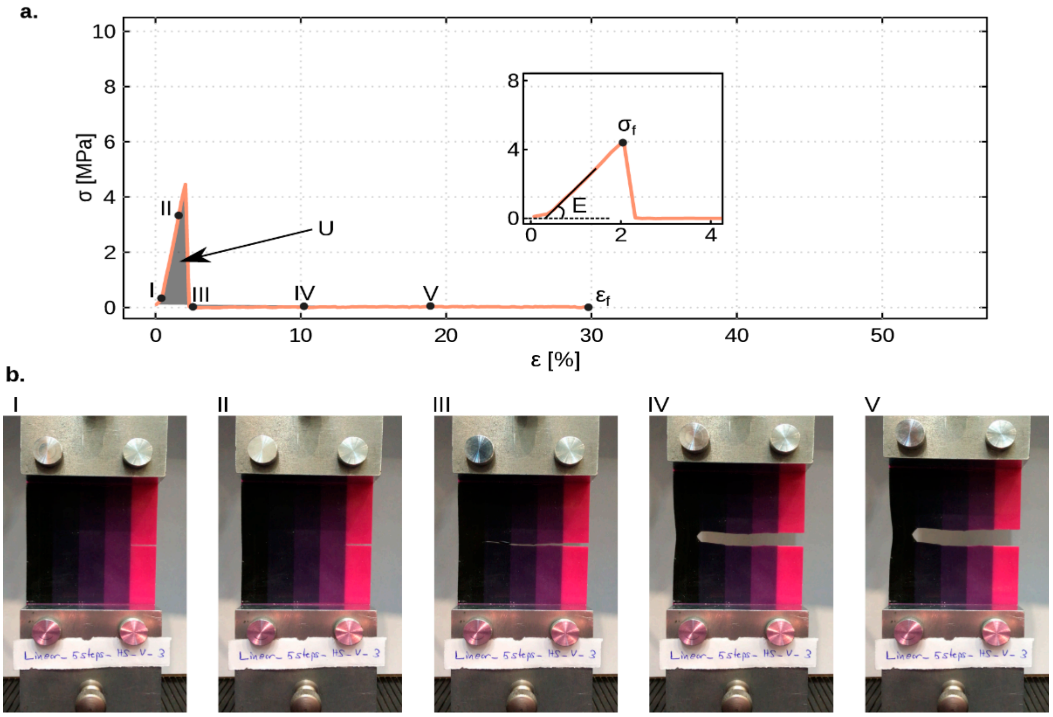

Figure S3. An illustration of the way the mechanical properties were calculated from the typical stress-strain curves of graded/non-graded specimens (a). The propagation of a crack initiated in the

hard (pink) phase to the soft (black) phase in a specimen with a 5-steps gradient and a transition length of 100%*W* (b). The initial crack swiftly propagated in the hard region (I-III), demonstrating the hallmarks of a brittle failure. The crack stopped at the soft region (IV) and exhibited a blunting zone at its tip during (V).
